# Association of 18F-fluorodeoxyglucose uptake with cardiac events in cardiac sarcoidosis during outpatient follow-up after immunosuppression

**DOI:** 10.1371/journal.pone.0347595

**Published:** 2026-05-14

**Authors:** Hideki Kawai, Masayoshi Sarai, Yasuchika Kato, Hiroyuki Naruse, Hiroshi Takahashi, Masakazu Tsujimoto, Kenta Nogami, Hiroshi Toyama, Shin-ichiro Morimoto, Hideo Izawa

**Affiliations:** 1 Department of Cardiology, Fujita Health University, Toyoake, Aichi, Japan; 2 Department of Medical Equipment Engineering, Fujita Health University, Toyoake, Aichi, Japan; 3 Department of Radiology, Fujita Health University Hospital, Toyoake, Aichi, Japan; 4 Department of Radiology, Fujita Health University, Toyoake, Aichi, Japan; Scuola Superiore Sant’Anna, ITALY

## Abstract

**Background:**

18F-fluorodeoxyglucose positron emission tomography/computed tomography (FDG-PET/CT) is a pivotal tool for diagnosing cardiac sarcoidosis, but its prognostic value during the phase of stable medical and device therapy after initiation of immunosuppressive therapy remains unclear. We aimed to evaluate the prognostic significance of cardiac FDG uptake in patients with cardiac sarcoidosis after treatment initiation.

**Methods:**

We retrospectively analyzed 79 patients who underwent FDG-PET/CT ≥ 12 months after initiating immunosuppressive therapy (June 2013–October 2023). Patients were categorized into the cardiac accumulation (+) and (-), and Cardiac metabolic activity (CMA) was also quantitatively measured. Major adverse cardiac events—including cardiac death, ventricular arrhythmias, ICD therapy, and heart failure hospitalization—were evaluated.

**Results:**

Patients in the cardiac accumulation (+) had a higher 2-year incidence of major adverse cardiac events than those in the cardiac accumulation (-), as determined by Kaplan–Meier analysis (log-rank P = 0.030), but FDG uptake was not identified as a predictor in Cox regression analysis. In long-term outcomes, the incidence of cardiac events tended to be higher in the cardiac accumulation (+) group, although this difference did not reach statistical significance (log-rank P = 0.078). Among patients with preserved left ventricular ejection fraction (LVEF ≥50%, independently associated with fewer events), annual cardiac event rates were similarly low regardless of uptake status (1.3% vs. 0.8%; log-rank P = 0.91). In 41 patients who underwent repeat PET imaging, CMA significantly decreased (median 4.83 to 0.82, P = 0.038). Among 23 patients without intensified immunosuppression despite uptake, it resolved spontaneously in 8 patients.

**Conclusions:**

Follow-up cardiac FDG uptake may be associated with an increased risk of short-term events but has limited value for predicting long-term prognosis. LVEF and the temporal dynamics of FDG uptake should be considered when managing cardiac sarcoidosis.

## Introduction

Sarcoidosis is characterized by non-caseating granulomas. Cardiac sarcoidosis is a concern owing to its potential to lead to irreversible left ventricular systolic dysfunction and life-threatening arrhythmias, making early detection and treatment crucial [1–5]. In patients with cardiac sarcoidosis, factors such as reduced left ventricular systolic function [6,7], ventricular arrhythmias [6], and extensive delayed enhancement on cardiac magnetic resonance imaging (MRI) [8] are established poor prognostic indicators.

18F-fluorodeoxyglucose-positron emission tomography/computed tomography (FDG-PET/CT) is crucial for detecting myocardial inflammation and has become a key diagnostic tool for cardiac sarcoidosis [9]. Regarding its prognostic value, studies have shown that patients with systemic sarcoidosis and cardiac FDG uptake have worse outcomes [10]. This has been corroborated by the findings of a recent meta-analysis of 55 studies involving over 5,000 patients, which confirmed the prognostic value of abnormal cardiac FDG uptake in sarcoidosis [11]. However, recent registry studies have questioned the association between FDG uptake and cardiovascular events [4,6], leading to a debate over the prognostic utility of FDG-PET in cardiac sarcoidosis. Additionally, there is limited research exploring the relationship between metabolic activity on follow-up PET and prognosis in patients with chronic phase after immunosuppressive therapy. Indeed, the 2024 consensus statements from both the American Heart Association and the European Society of Cardiology highlight the critical need for evidence to guide the management of patients with suspected or confirmed cardiac sarcoidosis, particularly regarding the interpretation of follow-up imaging and risk stratification during the chronic phase of the disease [3,5].

Here, we aimed to quantitatively assess metabolic activity on PET in patients with cardiac sarcoidosis during the phase of stable medical and device therapy (i.e., a period without recent clinical instability)—defined as at least 1 year after steroid initiation—and examine its association with future prognosis. In patients who underwent an additional FDG-PET/CT following the examined scan, a similar quantitative assessment was performed and the results were compared with the initial findings.

## Materials and methods

### Population

The study protocol was approved by the Fujita Health University Human Research Committee and conformed to the ethical guidelines of the 1975 Declaration of Helsinki. The requirement for informed consent was waived because of the retrospective study design, and an opt-out approach was used in accordance with institutional guidelines. All patients with cardiac sarcoidosis were diagnosed according to the JCS 2016 Guideline on Diagnosis and Treatment of Cardiac Sarcoidosis [9]. We included consecutive cases of FDG-PET/CT involving patients with cardiac sarcoidosis who had started immunosuppressive therapy, specifically those who underwent PET/CT at least 12 months after initiating immunosuppressive therapy between June 2013 and October 2023. Immunosuppressive therapy eligibility was determined based on the guidelines of the Japanese Circulation Society and treatment was administered following the established protocol [9]. Patients were excluded if they were considered to be in an unstable clinical phase, defined as those who, within 6 months prior to FDG-PET/CT, had (1) been hospitalized for heart failure or experienced sustained ventricular tachycardia or ventricular fibrillation, (2) received new or upgraded pacemaker implantation, or (3) had any changes in prednisolone dosage or started new immunosuppressive agents. These criteria were applied not to exclude high-risk patients, but to align the clinical phase at the time of imaging, as FDG-PET findings may be transiently influenced by recent acute events or treatment modifications.

The primary endpoint was a composite cardiovascular event, including cardiac death, sustained ventricular tachycardia/fibrillation, appropriate activation of an implantable defibrillator, and hospitalization for heart failure. The clinical data were accessed for research purposes between 01/09/2024 and 31/12/2024. The authors had access to information that could identify individual participants during data collection; however, all data were anonymized prior to analysis.

### FDG-PET/CT Protocol

FDG-PET/CT was performed using a dedicated PET-CT scanner (Biograph mCT X-4R; Siemens K.K., Tokyo, Japan). Patients were instructed to follow a low-carbohydrate, fat-rich, and protein-permitted diet the day before the examination, followed by fasting for at least 18 h before the procedure. CT images were acquired in 2-mm slices with a 0.35 helical pitch at 120 kV and an average of 82 mA, with auto exposure control with dose modulation (CARE Dose 4D) and a matrix of 512 × 512 pixels. Whole-body scans were performed 60 min after an intravenous injection of 185 MBq of 18F-FDG, followed by cardiac imaging with electrocardiogram gating to assess left ventricular function. FDG-PET data were collected in a three-dimensional mode for 2 min in each bed position. The FDG-PET data had a matrix of 200 × 200 pixels. CT and FDG-PET images were fused to match a matrix of 512 × 512 pixels [12,13].

### FDG-PET/CT analysis

#### Visual assessment (Group Definition).

The presence or absence of pathological FDG myocardial uptake was primarily determined by visual assessment by two independent readers (M.S. and H.K.) blinded to clinical information, with consensus reached in all cases. Diffuse or homogeneous FDG uptake patterns without focal accumulation were interpreted as physiological and were not classified as pathological uptake. Cases with insufficient suppression or ambiguous uptake patterns were also excluded if deemed physiological. Based on this visual assessment, patients were categorized into cardiac accumulation (+) and (-) groups.

### Quantitative assessment

Independent of the visual classification, myocardial 18F-FDG accumulation was quantitatively assessed using RAVAT software (Nihon Medi-Physics Co., Ltd., Tokyo, Japan). Volumes of interest (VOIs) were defined for both the heart and aorta in the PET images at the locations previously described [13–16]. An elliptical VOI was positioned extrinsically around the heart, and columnar VOIs were placed at three points along the inner wall of the descending aorta. These locations included the proximal descending thoracic aorta, upper edge of the liver, and upper portion of the common iliac artery bifurcation. The height of each aortic VOI was fixed at 10.0 mm (equivalent to 5 pixels), while the diameter was adjusted according to the aortic width. The mean standardized uptake values (SUVs) of the three aortic VOIs were measured and used as reference values. In accordance with our previous studies, a threshold of 1.4 times the mean SUV of the descending aorta was used to identify high myocardial 18F-FDG accumulation [13–16]. Physiological myocardial uptake was carefully excluded; diffuse or homogeneous FDG uptake patterns without focal accumulation were interpreted as physiological and were not classified as pathological uptake. Consequently, only focal or focal-on-diffuse uptake exceeding the 1.4-fold aortic SUVmean threshold was included in the calculation of the cardiac metabolic volume (CMV). Cardiac metabolic activity (CMA) was then calculated by multiplying the CMV by the corresponding average SUV [13,17]. In this study, CMA refers to left ventricular values unless otherwise specified.

### Composite cardiac events

A major cardiac event was defined as a composite of cardiac death, sustained ventricular tachycardia, ventricular fibrillation, appropriate activation of an implantable cardioverter defibrillator, and hospitalization for heart failure. LVEF was assessed using transthoracic echocardiography performed as part of standard clinical evaluation. Follow-up data were collected by observers, who were blinded to the FDG-PET results, during clinical visits or standardized telephone interviews.

### Statistical analysis

The Shapiro–Wilk test was applied to evaluate the normality of continuous variables. Continuous variables are presented as mean ± standard deviation if normally distributed or as median (interquartile range) if not normally distributed. To compare intergroup differences, Student’s *t*-test was used for parametric variables and the Mann–Whitney U test for nonparametric variables. For categorical variables, the chi-squared test or Fisher’s exact test was employed. The Wilcoxon signed-rank test was used to assess within-subject changes. Cumulative survival curves were generated using Kaplan–Meier analysis, with the time from the initial FDG-PET/CT to the first event. Patients were categorized into the cardiac accumulation (+) and (-). Differences between time-to-event curves were analyzed using log-rank tests. The event rates for the cardiac accumulation (+) and (-) were compared using the Kaplan–Meier estimator. Univariate Cox proportional hazards models were used to assess predictors of cardiac events. Variables with a significance level of P < 0.05 in the univariate analyses, along with age and sex, were included in multivariable analysis. In the short-term analysis, troponin I was included in the multivariable model because it was statistically significant in the univariate analysis. The proportional hazards assumption was assessed using Schoenfeld residuals and log-minus-log survival plots, and no significant violations were observed. Wilcoxon signed-rank tests were used to analyze changes in quantitative cardiac uptake values in patients who underwent multiple FDG-PET scans. The results are presented as hazard ratios (HRs) with 95% confidence intervals (CIs). All statistical analyses were conducted using JMP Pro software (version 17.0, SAS Institute), with statistical significance set at P < 0.05.

## Results

### Patient Characteristics

The median age of the 79 patients was 67 (range 57–72) years, with 53 (67%) being women. Patients were categorized into the cardiac accumulation (+) and (-). Significant differences were observed in CMA (P < 0.0001), FDG accumulation in the right ventricle (P = 0.0001) and extramyocardial areas (P = 0.017) between the groups. The median prednisolone dose was 5 mg in both groups, with methotrexate used in one patient from each group (Table 1).

**Table 1:** Patient characteristics.

	Cardiac accumulation (+) (n = 38)	Cardiac accumulation (-) (n = 41)	P-value
Age (years)	67 (57, 73)	65 (59, 72)	0.73
Female sex, n (%)	28 (73.7%)	25 (61.0%)	0.23
Isolated cardiac sarcoidosis, n (%)	7 (18.4%)	5 (12.2%)	0.44
ACE (U/L)	12.3 (10.6, 15.4)	12.4 (9.1, 14.7)	0.43
sIL-2R (U/mL)	332 (239, 513)	355 (269, 461)	0.51
Troponin I (ng/mL)	0.006 (0.004, 0.014)	0.006 (0.004, 0.011)	0.85
NT-proBNP (pg/mL)	291 (76, 977)	329 (143, 683)	0.87
Sustained VT/VF, n (%)	5 (13.2%)	4 (9.8%)	0.63
Advanced atrioventricular block, n (%)	21 (58.3%)	27 (67.5%)	0.41
Abnormal left ventricular morphology (basal septal thinning or ventricular aneurysm), n (%)	23 (60.5%)	22 (53.7%)	0.54
LV dysfunction (ejection fraction<50%) or local LV wall motion abnormality, n (%)	24 (63.2%)	21 (51.2%)	0.28
Delayed contrast enhancement of myocardium on gadolinium-enhanced MRI, n (%)	24/28 (85.7%)	19/24 (79.2%)	0.53
CMA	66.2 (17.2, 300.0)	0.24 (0.022, 1.60)	<0.0001
Cardiac accumulation of FDG in RV, n (%)	9 (23.7%)	0 (0%)	0.0001
Extra-cardiac accumulation of FDG, n (%)	25 (65.8%)	16 (39.0%)	0.017
Prednisolone dosage (mg)	5 (5, 5)	5 (5, 5)	0.31
Concomitant methotrexate use, n (%)	1 (2.6%)	1 (2.6%)	0.99

Values are presented as median (interquartile range) and number of patients (percentage).

ACE: angiotensin-converting enzyme, FDG: 18F-fluorodeoxyglucose, LV: left ventricle, MRI: magnetic resonance imaging, NT-proBNP: N-terminal pro–brain natriuretic peptide, RV: right ventricle, sIL-2R: soluble interleukin-2 receptor, VF: ventricular fibrillation, VT: ventricular tachycardia.

### Clinical outcomes

Cardiac events by group are summarized in Table 2. Over a median follow-up of 4 years, 9 patients in the cardiac accumulation (+) experienced cardiac events, with seven of these events occurring within 2 years post FDG-PET. In the cardiac accumulation (-), 4 patients had cardiac events, with only one event occurring within the first 2 years.

**Table 2:** Major cardiovascular events after FDG-PET/CT during the phase of stable medical and device therapy.

	Cases	Cardiac accumulation (+) (n = 38)	Cardiac accumulation (-) (n = 41)
		No. of events (No. within 2 years)	No. of events (No. within 2 years)
Observational period, months		44.6 (23.4, 78.7)	64.3 (26.1, 92.2)
Composite event, n (within 2 years)	13 (8)	9 (7)	4 (1)
Cardiac death	2	1	1
Sustained VT/VF or appropriate activation of ICD	10 (6)	8 (5)	2 (1)
Hospitalization for heart failure	5 (4)	4 (3)	1

**Fig 1:**
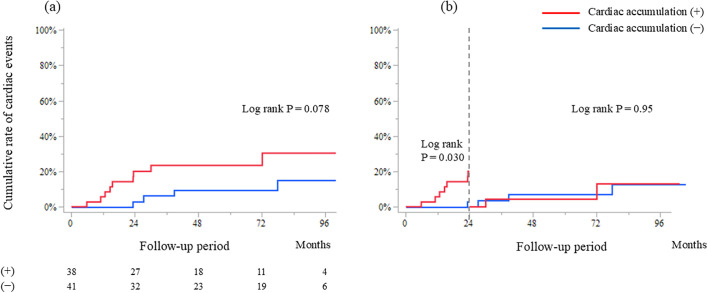
Composite of cardiac events after FDG-PET/CT. (a) Kaplan–Meier curves for the entire follow-up period. (b) Comparisons of events within the first 2 years and after 2 years post FDG-PET/CT. Vertical dashed line indicates the 2-year follow-up point. P values calculated using log-rank test. FDG-PET/CT, ^18^F-fluorodeoxyglucose-positron emission tomography/computed tomography. Supplementary S1 Fig Association between composite cardiac events and cardiac metabolic activity (CMA).

The Kaplan–Meier analysis showed no significant difference in composite cardiac events between the cardiac accumulation (+) and (-) over the entire follow-up period (log-rank P = 0.078). However, within the first 2 years, the outcomes were significantly worse in the cardiac accumulation (+) than in the cardiac accumulation (-) (log-rank P = 0.030) (Fig 1). Supplemental analyses using SUVmax, SUVmean, and CMV showed trends consistent with the CMA-based results, and none of these parameters predicted short-term or long-term outcomes in Cox regression models. These findings indicate that the choice of CMA as the primary quantitative index did not alter the overall conclusions of the study. When the cardiac accumulation (+) was further stratified into three subgroups based on CMA tertiles, the incidence of long-term events did not show a stepwise increase with higher CMA values. Specifically, the cumulative cardiac event rates were similar between the middle (CMA 30–199) and highest (CMA ≥ 200) CMA tertiles, both of which were higher than the rate observed in the lowest CMA tertile (CMA < 30). (Supplementary S1 Fig).

Table 3 presents the Cox proportional hazards analyses for major cardiac events. In the univariate analyses, long-term composite cardiac events were associated with cardiac troponin I levels, sustained VT/VF, and LV dysfunction, but not with the presence of LV accumulation (P = 0.077), LV-CMA as a continuous variable (P = 0.78), or log-transformed LV-CMA on PET (P = 0.46). In the multivariate analysis adjusted for age, sex, and sustained VT/VF, LV dysfunction (EF < 50%) was an independent predictor (HR 5.22, 95% CI 1.08–25.43, P = 0.040) as well as cardiac troponin I level (HR 1.03, 95% CI 1.00–1.08, P = 0.034) (Table 3A). In the short-term analysis, only cardiac troponin I level was predictive in univariate analysis (HR 1.04, 95% CI 1.00–1.07, P = 0.045), whereas the presence of LV accumulation (P = 0.064), LV-CMA as a continuous variable (P = 0.81), and log-transformed LV-CMA on PET (P = 0.16) were not. In the multivariate analysis adjusted for age and sex, cardiac troponin I remained an independent predictor (HR 1.05, 95% CI 1.00–1.08, P = 0.029) (Table 3B).

**Table 3:** Cox analysis for major cardiac events in the (A) long-term and at (B) 2-year follow-ups.

(A) Long term						
	Univariate analysis			Multivariate analysis		
	HR	95% CI	P-value	HR	95% CI	P-value
Age (years)	1.03	0.98–1.09	0.32	1.03	0.98–1.09	0.31
Female sex	0.37	0.12–1.10	0.07	0.60	0.19–1.87	0.38
Isolated CS	1.14	0.25–5.16	0.86			
ACE (U/L)	1.07	0.94–1.18	0.24			
sIL-2R (U/mL)	1.00	0.99–1.00	0.37			
Troponin I (ng/mL) (0.01 ng/mL increase)	1.04	1.00–1.07	0.034	1.03	1.00–1.08	0.034
NT-pro BNP (pg/mL) (100 pg/mL increase)	1.04	0.98–1.08	0.086			
Sustained VT/VF	4.78	1.56–14.64	0.013	2.58	0.79–8.40	0.12
Advanced atrioventricular block	0.64	0.20–2.03	0.46			
Abnormal LV morphology (basal septal thinning or ventricular aneurysm)	1.00	0.32–3.08	0.99			
LV dysfunction (ejection fraction<50%)	7.17	1.59–32.36	0.010	5.22	1.08–25.43	0.040
LGE on MRI	NA					
LV accumulation on PET	2.77	0.85–9.02	0.077			
LV-SUVmean on PET (continuous value)	0.94	0.35–1.76	0.87			
LV-SUVmax on PET (continuous value)	1.00	0.80–1.14	0.98			
LV-CMV on PET (continuous value)	1.00	0.99–1.01	0.57			
LV-CMA on PET (continuous value)	1.00	1.00–1.00	0.78			
Log LV-CMA on PET	1.15	0.80–1.69	0.46			
RV cardiac uptake on PET	1.51	0.33–6.85	0.59			
(B) 2-year follow-up						
	Univariate analysis			Multivariate analysis		
	HR	95% CI	P-value	HR	95% CI	P-value
Age (years)	1.02	0.96–1.09	0.60	1.02	0.95–1.13	0.56
Female sex	0.79	0.19–3.33	0.76	0.32	0.05–1.90	0.21
Isolated CS	0.84	0.10–6.86	0.87			
ACE (U/L)	1.14	0.99–1.27	0.066			
sIL-2R (U/mL)	1	0.99–1.00	0.38			
Troponin I (ng/mL) (0.01 ng/mL increase)	1.04	1.00–1.07	0.045	1.05	1.00–1.08	0.029
NT-pro BNP (pg/mL) (100 pg/mL increase)	1.04	0.98–1.08	0.19			
Sustained VT/VF	4.22	1.01–17.69	0.070			
Advanced atrioventricular block	2.57	0.57–11.49	0.22			
Abnormal LV morphology (basal septal thinning or ventricular aneurysm)	1.42	0.36–5.70	0.62			
LV dysfunction (ejection fraction<50%)	3.77	0.76–18.66	0.10			
LGE on MRI	NA					
LV accumulation on PET	7.27	0.89–59.09	0.064			
LV-SUVmean on PET (continuous value)	0.91	0.26–1.87	0.85			
LV-SUVmax on PET (continuous value)	1.01	0.78–1.17	0.88			
LV-CMV on PET (continuous value)	1.00	0.99–1.02	0.52			
LV-CMA on PET (continuous value)	1.00	1.00–1.00	0.81			
Log LV-CMA on PET	1.41	0.88–2.48	0.16			
RV cardiac uptake on PET	2.47	0.50–12.3	0.27			

ACE: angiotensin-converting enzyme, CI: confidence interval, CMA: cardiac metabolic activity, CS: cardiac sarcoidosis, HR: hazard ratio, LGE: late gadolinium enhancement, LV: left ventricle, MRI: magnetic resonance imaging, NT-proBNP: N-terminal pro–brain natriuretic peptide, sIL-2R: soluble interleukin-2 receptor, Trp I: Troponin I, VF: ventricular fibrillation, VT: ventricular tachycardia

**Fig 2:**
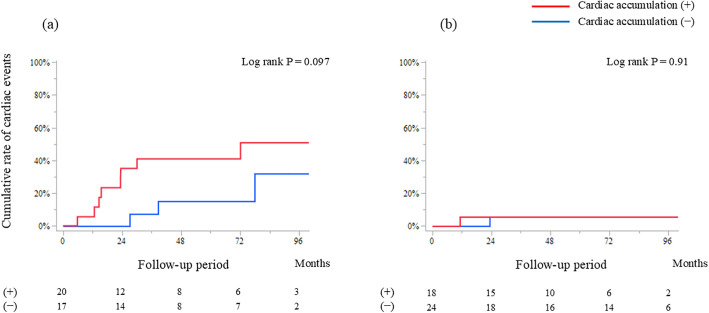
Composite of cardiac events after FDG-PET/CT stratified by baseline left ventricular ejection fraction (LVEF). (a) LVEF<50% and **(b)** LVEF≥50%. Vertical dashed line indicates the 2-year follow-up point. P values calculated using log-rank test. FDG-PET/CT, ^18^F-fluorodeoxyglucose-positron emission tomography/computed tomography; LVEF, left ventricular ejection fraction.

In patients with LVEF < 50% (n = 37), the incidence of cardiac events (n = 11) tended to be higher in the cardiac accumulation (+) group than in the (-) group, although this difference did not reach statistical significance (log-rank P = 0.097). In patients with LVEF≥50% (n = 42), cardiac events occurred in just 2 patients. The rates of composite cardiac events were also similar between the cardiac accumulation (+) and (-) groups (annual cardiac event rates 1.3% vs. 0.8%; log-rank P = 0.91) (Fig 2).

Observational periods are presented as median (interquartile range). Values are number of patients; numbers in parentheses indicate events within 2 years after FDG-PET/CT. FDG-PET/CT: 18F-fluorodeoxyglucose-positron emission tomography/computed tomography, ICD: implantable cardioverter defibrillator, LVDd: left ventricular end-diastolic dimension, LVEF: left ventricular ejection fraction, VF: ventricular fibrillation, VT: ventricular tachycardia

### Post-FDG-PET Strategy and Cardiac Uptake Changes

**Fig 3:**
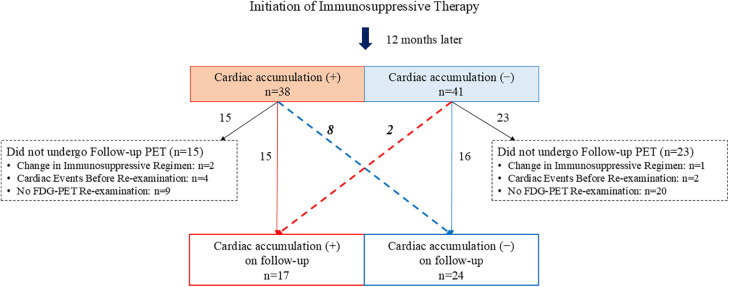
Post-FDG-PET/CT clinical management strategy and changes in cardiac uptake between baseline and additional scans. CMA, cardiac metabolic activity; FDG-PET/CT, ^18^F-fluorodeoxyglucose-positron emission tomography/computed tomography.

**Fig 4:**
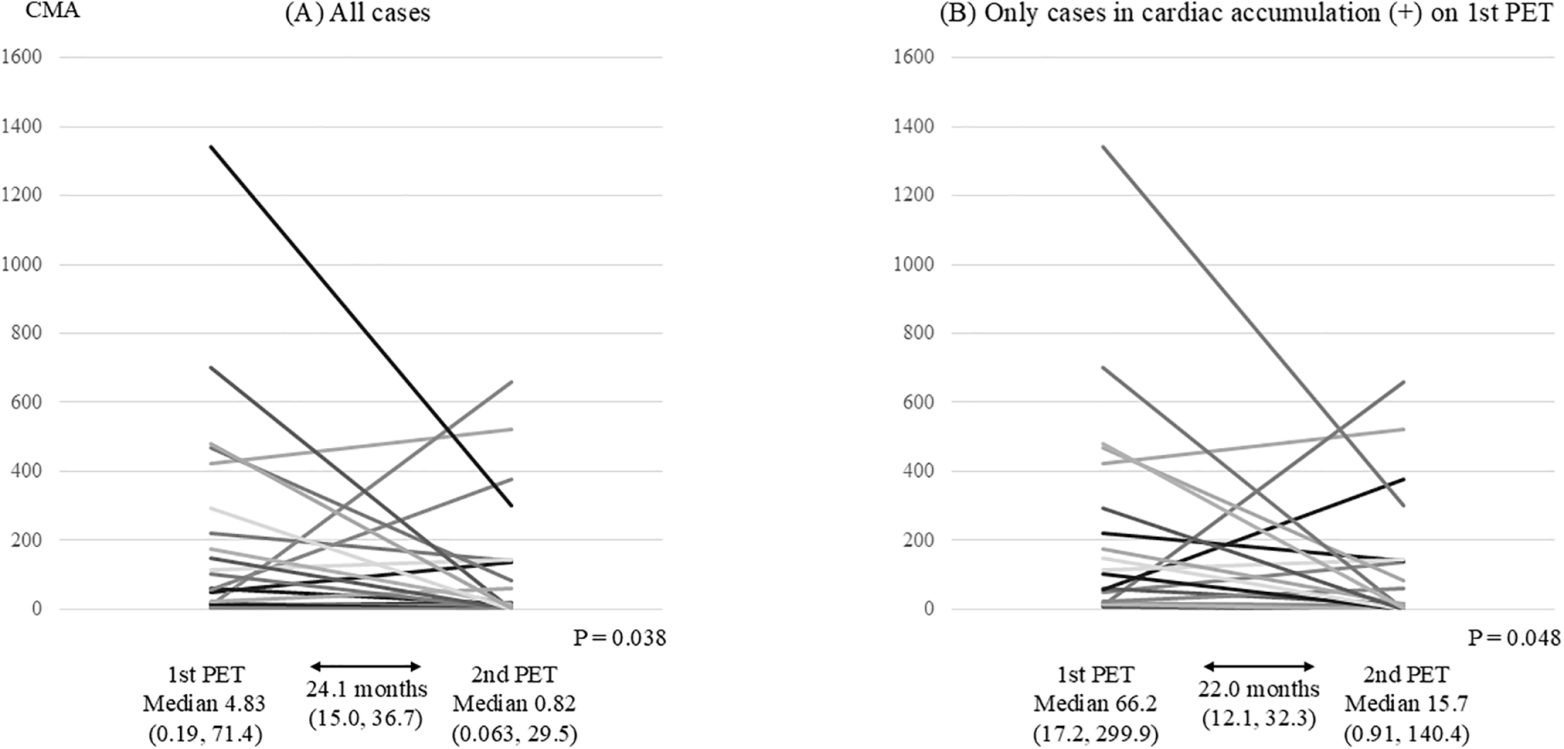
Quantitative cardiac uptake values in consecutive FDG-PET/CT scans. CMA values are expressed in **g·**SUV (CMA = cardiac metabolic volume × mean standardized uptake value). FDG-PET/CT, ^18^F-fluorodeoxyglucose-positron emission tomography/computed tomography.

Among the 38 patients in the cardiac accumulation (+), the immunosuppressive regimen was changed in two patients, cardiac events occurred before FDG-PET re-examination in four patients, and re-examination was not performed in 9 patients. Of the remaining 23 patients, 15 showed the cardiac accumulation on follow-up, whereas 8 did not. In the cardiac accumulation (-) (41 patients), immunosuppressive regimens were changed in 1 patient, cardiac events occurred before re-examination in 2 patients, and re-examination was not performed in 20 patients. Of the other 18 patients, 2 developed cardiac uptake, while 16 remained without cardiac uptake on FDG-PET re-examination (Fig 3). The follow-up CMA decreased from 4.83 to 0.82 (P = 0.038), and in patients with initial follow-up cardiac uptake, CMA also decreased from 66.2 to 15.7 (P = 0.048) (Fig 4).

### Representative Cases

**Fig 5:**
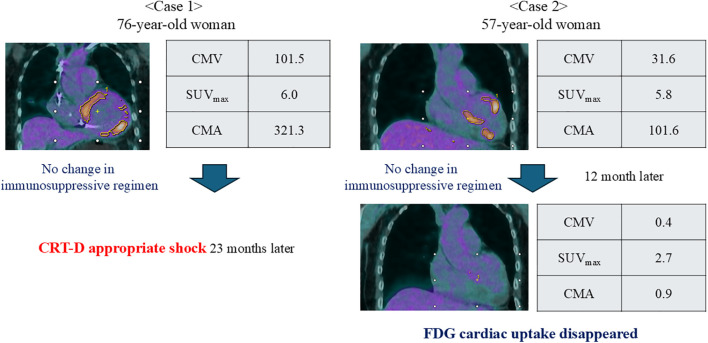
Representative cases of patients with cardiac uptake. Both cases demonstrated FDG cardiac uptake during the phase of stable medical and device therapy; however, their subsequent clinical courses differed substantially. **Case 1:** A 76-year-old woman with sustained ventricular tachycardia (VT), reduced left ventricular ejection fraction (LVEF = 22%), and an aneurysm in the left ventricular apex was diagnosed with systemic sarcoidosis based on lung tissue biopsy findings. She underwent implantation of a cardiac resynchronization therapy device with defibrillator (CRT-D). FDG-PET was performed 2 years after the initiation of corticosteroid therapy. Twenty-three months after therapy, CRT-D was appropriately activated in response to sustained VT. **Case 2:** A 57-year-old woman with non-sustained VT, mildly reduced LVEF (48%), and basal septal thinning was diagnosed with isolated cardiac sarcoidosis through additional evaluations using cardiac MRI and FDG-PET/CT. One year after the initiation of corticosteroid therapy, FDG-PET was performed. At a follow-up examination 12 months later, the FDG cardiac uptake had resolved. CMV, cardiac metabolic volume; CMA, cardiac metabolic activity; FDG-PET/CT, 18F-fluorodeoxyglucose-positron emission tomography/computed tomography; MRI, magnetic resonance imaging.

Case 1 involved a 76-year-old woman who presented with a CMV of 101.5, SUV_max_ of 6.0, and CMA of 321.3. She developed appropriate shock from implantation of a cardiac resynchronization therapy device using a defibrillator 23 months later. Case 2 involved a 57-year-old woman with a CMV of 31.6, SUV_max_ of 5.8, and CMA of 101.6 who showed no cardiac uptake on FDG-PET re-examination after 12 months, with reduced values (CMV of 0.4, SUV_max_ of 2.7, and CMA of 0.9) (Fig 5).

## Discussion

In 79 patients with cardiac sarcoidosis who underwent FDG-PET after starting corticosteroid treatment during the phase of stable medical and device therapy, 13 composite events occurred over a median follow-up of 4 years. When patients were categorized into cardiac accumulation (+) and (-), cardiac accumulation was significantly associated with a higher incidence of cardiac events within the first 2 years. However, when this metabolic activity was assessed as a continuous quantitative variable, it did not emerge as a statistically significant predictor of short-term events in the Cox analysis. For the overall long-term prognosis, the most robust independent predictor was not inflammatory activity but rather established myocardial damage, such as LVEF<50% and elevated cardiac troponin levels. For patients with LVEF≥50%, the annual incidence of cardiac events was very low (0.8%–1.3%) in both groups. Additionally, 41 of the 79 patients underwent repeat FDG-PET imaging. Of the 23 patients in the cardiac accumulation (+), 8 showed low values on repeat imaging. Overall, CMA values significantly decreased upon repeat imaging, even in patients with initially high CMA values. This reduction should not be interpreted solely as improvement of inflammatory activity, as FDG uptake may also decline due to loss of viable myocardium or progression of irreversible myocardial injury. Without perfusion or fibrosis imaging, these mechanisms cannot be distinguished. Nevertheless, most patients with decreased CMA did not experience cardiac events or worsening left ventricular function, suggesting that such metabolic changes may not necessarily correspond to clinically significant deterioration. These considerations highlight that variations in CMA should be interpreted cautiously and in conjunction with clinical status. These interpretations lead us to propose the following clinical implications and hypotheses:

1.Patients with cardiac FDG uptake during the phase of stable medical and device therapy after corticosteroid initiation may have a long-term prognosis similar to those without uptake but may need closer monitoring in the short term.
2.Some patients with high-CMA showed a decrease in FDG uptake without changes to their immunosuppressive regimens, suggesting that FDG uptake is dynamic and reversible. Given the potential for spontaneous improvement, caution should be exercised when considering intensified immunosuppressive therapy owing to potential side effects.
3.In patients with normal LVEF, the incidence of cardiac events was very low regardless of the CMA, suggesting that concerns about cardiac events may be unwarranted in this subgroup.

This study’s central finding—that FDG uptake in the phase of stable medical and device therapy after immunosuppressive therapy did not independently predict long-term outcomes, which were instead driven by markers of established myocardial damage such as LVEF—aligns with recent findings in this field. Okafor et al. investigated serial FDG and Rubidium-82 PET in 113 patients and concluded that post-treatment risk was determined by the extent of perfusion defect (scar), rather than residual myocardial inflammation [18]. They argued that while suppressing inflammation is the goal of therapy, the subsequent risk is dictated by the remaining scar burden. This argument provides independent validation for our conclusion that in the chronic phase of stable medical and device therapy for cardiac sarcoidosis, assessing the degree of irreversible cardiac damage is paramount for long-term risk stratification. Although multiple PET-based quantitative indices can be evaluated, including SUVmax and CMV, our supplemental analyses demonstrated that these parameters yielded results consistent with those obtained using CMA. Therefore, focusing on CMA did not alter the overall interpretation of the prognostic implications.

### Previous Studies on Prognostic Roles of FDG-PET

FDG-PET is valuable for detecting myocardial inflammation in cardiac sarcoidosis, a condition often difficult to diagnose through biopsy [19]. However, its role in assessing treatment response and predicting prognosis remains debated. A few studies have investigated the prognostic role of cardiac PET in sarcoidosis, and they may lack uniform patient backgrounds [11]. A meta-analysis identified six retrospective studies, but these studies often involved patients with suspected cardiac sarcoidosis or those already undergoing immunosuppressive therapy. The results typically showed worse outcomes in patients with cardiac FDG uptake, but this was expected as most patients in the uptake group had cardiac sarcoidosis, whereas many in the non-uptake group did not.

In a study by Gowani et al., 50 patients with cardiac sarcoidosis who underwent both cardiac MRI and FDG-PET/CT were evaluated. They found that late gadolinium enhancement on MRI was prognostically useful, but residual FDG uptake on PET was not [20]. Similarly, a large study in Japan concluded that the degree of FDG uptake was not linked to prognosis [6]. A few studies have quantitatively assessed FDG-PET findings before and after immunosuppressive therapy, though most involved fewer than 20 cases [21–23]. Rojulpote et al. analyzed FDG-PET findings of 83 patients with cardiac sarcoidosis and found that the treatment response varied, with no significant link between PET response and prognosis, which aligns with our findings. However, their study had limitations, including variability in immunosuppressive therapy doses and timing of FDG-PET scans [24]. Kaneta et al. qualitatively evaluated 80 patients with cardiac sarcoidosis (68 with PET and 12 with gallium scintigraphy) and reported that recurrent radiologic activity was frequently observed during follow-up. They also demonstrated that intensified immunosuppressive therapy suppressed this recurrent activity and was associated with more favorable outcomes in selected patients with active inflammation. However, our clinical approach differs in that we do not routinely intensify immunosuppression solely on the basis of residual FDG uptake during the phase of stable medical and device therapy. In patients without high-risk features—such as reduced LVEF, progressive ventricular dysfunction, or malignant arrhythmias—the potential risks of excessive immunosuppression, including infection and steroid-related adverse effects, must be carefully considered. Our findings suggest that, in the phase of stable medical and device therapy for cardiac sarcoidosis, long-term prognosis is largely determined by irreversible myocardial damage rather than by residual metabolic activity alone. Accordingly, treatment escalation should be individualized rather than uniformly applied to all patients with residual FDG uptake [25]. Imamura et al. showed that higher SUVmax after immunosuppressive therapy and impaired left ventricular ejection fraction predicted adverse outcomes in cardiac sarcoidosis. While their study incorporated serial pretreatment and follow-up FDG-PET data, our study focused on patients who appeared clinically stable on medical and device therapy during outpatient follow-up and did not include pretreatment imaging [26]. Our cohort included patients diagnosed before FDG-PET became widely used in clinical practice, allowing inclusion of a larger single-center cohort and detailed quantitative assessment of myocardial FDG uptake on follow-up FDG-PET. Despite differences in study design, both studies consistently identified impaired left ventricular systolic function as a major prognostic determinant, and prior long-term data indicate that patients clinically stable on medical and device therapy remain at substantial risk.

### Physiological Implications of FDG Uptake in the Phase of Stable Medical and Device Therapy after Initiating Immunosuppressive Therapy

A central question in our study is the physiological meaning of metabolic activity observed on follow-up PET. Generally, this is thought to reflect persistent active granulomatous inflammation. However, while FDG is a marker for the enhanced glucose utilization characteristic of inflammatory cells, it is known that hibernating or stunned myocardium can also exhibit increased glucose uptake. Therefore, it is possible that the observed activity reflects a non-specific metabolic shift in scarred or dysfunctional myocardial tissue. Distinguishing between these possibilities could be facilitated by complementary imaging with iodine-123-beta-methyl-iodophenyl-pentadecanoic acid (¹²³I-BMIPP) scintigraphy, which assesses fatty acid metabolism, although this was not performed in our cohort.

Nevertheless, several factors support the interpretation of residual uptake as primarily inflammatory. For instance, it has been reported that a reduction in FDG uptake was associated with an improvement in LVEF, suggesting that FDG activity is linked to a reversible pathological process such as inflammation rather than a fixed scar [27]. This aligns with our observation, that is, a significant number of patients showed spontaneous resolution of FDG uptake without treatment escalation. Collectively, our findings suggest that inflammatory activity in stable CS can be dynamic, with the potential to wane over time even under low-dose maintenance therapy.

Our finding of risk dissociation, where FDG uptake suggests short-term but not long-term events, may reflect the different pathophysiological roles of active inflammation and established scar. We hypothesize that the observed residual metabolic activity represents a non-progressive, smoldering inflammation. This inflammation may be insufficient to drive long-term ventricular remodeling but could transiently increase myocardial instability, predisposing patients to adverse cardiac events such as arrhythmias and heart failure exacerbations in the short term. The spontaneous resolution of FDG uptake observed in a significant portion of our cohort supports this hypothesis of a dynamic self-limiting inflammatory state, distinct from the fixed scar-mediated risk that dictates long-term prognosis.

### Definition of Relapse and Treatment Strategies for Cardiac Sarcoidosis

An international survey on the treatment of cardiac sarcoidosis revealed significant variability in corticosteroid management strategies, such as dosing, tapering, duration, and adjunctive immunosuppressive agents [28]. This reflects differences in treatment approaches across institutions. The definition of relapse and how to adjust immunosuppressive therapy in such cases also varies widely [28]. At our institution, metabolic activity on PET during the phase of stable medical and device therapy after initiating immunosuppressive therapy does not lead to dosage increase or addition of other immunosuppressive agents. Instead, therapy intensification is based on factors such as LV function and life-threatening ventricular arrhythmias. Even in patients with resolved FDG uptake and stable clinical courses, corticosteroids are maintained as lifelong therapy. Our findings support this conservative approach in patients clinically stable on medical and device therapy, though further prospective validation is warranted.

This study has certain limitations. First, it was a single-center retrospective analysis, which limits the generalizability of the findings. A multicenter prospective study is needed to validate and expand these results, potentially providing a broader dataset and stronger evidence for clinical decision-making. Second, the relatively small sample size (n = 79) may reduce the robustness of the multivariable analysis and increase the risk of overfitting. Therefore, the results should be interpreted as exploratory and require validation in larger cohorts. Third, pre-treatment FDG-PET was not uniformly available because a subset of patients were diagnosed before FDG-PET/CT became available and were evaluated with gallium scintigraphy instead. Therefore, interval changes in FDG uptake from treatment initiation could not be systematically assessed, limiting interpretation regarding treatment response or relapse. However, the primary aim of this study was not to evaluate treatment response but to assess the prognostic significance of FDG uptake during the phase of stable medical and device therapy (≥12 months after treatment initiation); thus, the absence of baseline FDG-PET does not affect the validity of our main findings. Furthermore, we acknowledge that the use of echocardiography for evaluating longitudinal changes in left ventricular function is subject to inter-observer variability, and cardiac MRI is generally considered a more objective modality. However, restricting our analysis solely to MRI-derived parameters was not feasible in this real-world cohort. A substantial proportion of our patients had advanced conduction abnormalities or severe arrhythmias requiring implanted cardiac devices (e.g., pacemakers and ICDs), which often preclude serial MRI assessments or result in non-diagnostic image quality due to artifacts. Because transthoracic echocardiography remains the standard, guideline-recommended modality for routine longitudinal follow-up, its inclusion in our composite endpoint was essential to accurately reflect real-world clinical disease progression and management. Finally, we acknowledge the potential for immortal time bias inherent to our study design. As all patients were required to undergo FDG-PET during the phase of stable medical and device therapy (≥12 months after initiating immunosuppressive therapy), those who experienced early adverse events or were unable to complete imaging were inherently excluded. This selection process may have led to an underestimation of event rates in higher-risk patients and limits generalizability.

Nonetheless, this design enabled us to specifically examine the clinical significance of FDG uptake during the post-treatment phase of stable medical and device therapy, which remains a relevant but underexplored area. These limitations underscore the need for future research with standardized imaging protocols before and after immunosuppressive therapy, as well as consideration of institutional differences in imaging modalities and therapeutic strategies.

## Conclusions

In the phase of stable medical and device therapy after initiating immunosuppressive therapy, high cardiac FDG uptake was associated with a higher short-term incidence of cardiac events but was not an independent predictor, and quantitative CMA was not significantly associated with prognosis. Long-term outcomes were not significantly affected by uptake status, and the risk of events remained very low in patients without markers of significant myocardial damage, such as left ventricular systolic dysfunction or elevated cardiac troponin levels. Given the dynamic nature of FDG uptake and the potential for spontaneous improvement, uptake findings should be interpreted in conjunction with other clinical parameters rather than used as the sole basis for intensifying immunosuppressive therapy, to avoid overtreatment.

## Supporting information

S1 Fig Association between composite cardiac events and CMA. (TIF)

S1 Table Age-adjusted Cox proportional hazards models. Hazard ratios adjusted for age.
(DOCX)
