# Supplementary figures and images for: Association of 18F-fluorodeoxyglucose uptake with cardiac events in cardiac sarcoidosis during outpatient follow-up after immunosuppression
Source: PLoS One. 2026 May 14;21(5):e0347595. doi: 10.1371/journal.pone.0347595 (PMC13175335; doi:10.1371/journal.pone.0347595)

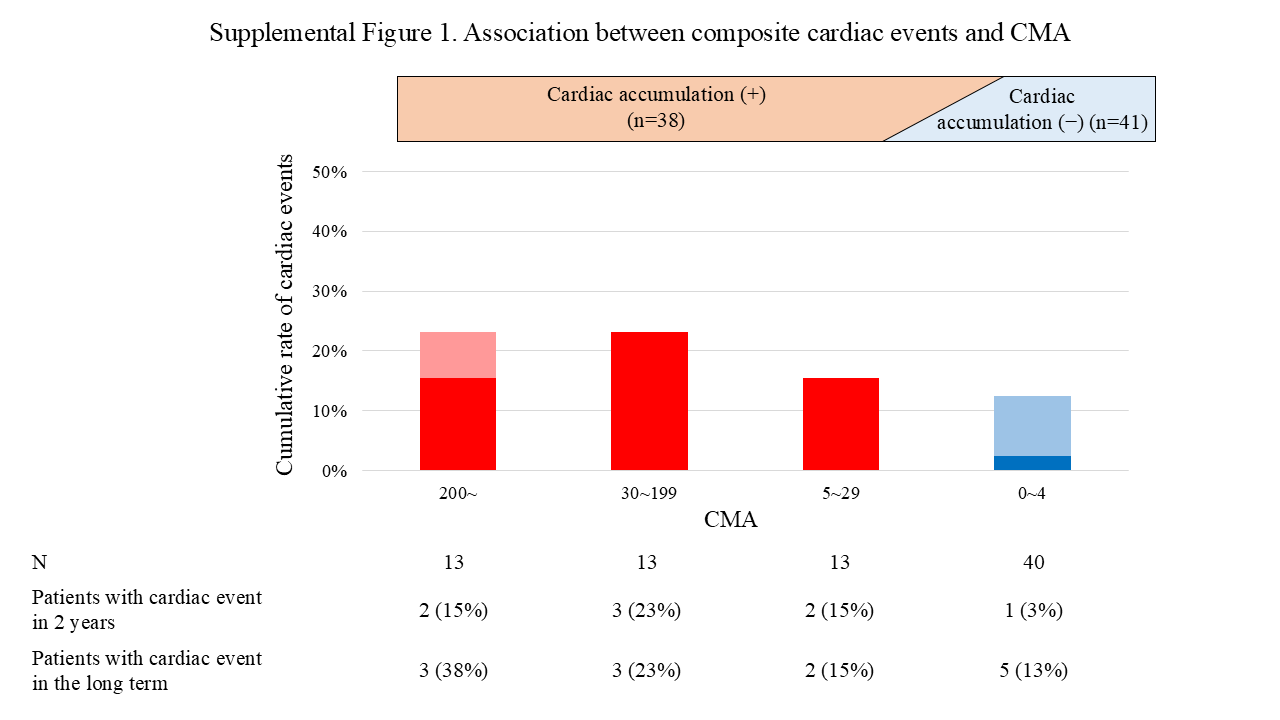

Supplement: S1 Fig — (TIF) [file pone.0347595.s001.tif]
