# Supplementary material for: Association of 18F-fluorodeoxyglucose uptake with cardiac events in cardiac sarcoidosis during outpatient follow-up after immunosuppression
Source: PLoS One. 2026 May 14;21(5):e0347595. doi: 10.1371/journal.pone.0347595 (PMC13175335; doi:10.1371/journal.pone.0347595)
Supplement: S1 Table — Hazard ratios adjusted for age. (DOCX) [file pone.0347595.s002.docx]

**Supporting Information**

**S1 Table. Age-adjusted Cox proportional hazards models.**

**Hazard ratios adjusted for age.**

| (A) Long term | Unadjusted | | | Age-adjusted | | |
| --- | --- | --- | --- | --- | --- | --- |
|  | HR | 95% CI | P-value | HR | 95% CI | P-value |
| Trp I (ng/mL) (100 ng/mL increase) | 1.04 | 1.00–1.07 | 0.034 | 1.04 | 1.00–1.07 | 0.033 |
| NT-pro BNP (pg/mL) (0.01 pg/mL increase) | 1.04 | 0.98–1.08 | 0.086 | 1.03 | 0.98–1.08 | 0.16 |
| Sustained VT/VF | 4.78 | 1.56–14.64 | 0.013 | 4.74 | 1.54–14.57 | 0.013 |
| LV dysfunction (ejection fraction<50%) | 7.17 | 1.59–32.36 | 0.010 | 7.64 | 1.69–34.66 | 0.008 |
| LV-CMA on PET (continuous value) | 1.00 | 1.00–1.00 | 0.78 | 1.00 | 1.00–1.00 | 0.79 |
| Log LV-CMA on PET | 1.15 | 0.80–1.69 | 0.46 | 1.11 | 0.73–1.69 | 0.62 |
|  |  |  |  |  |  |  |
| (B) 2-year follow-up |  |  |  |  |  |  |
| Trp I (ng/mL) (100 ng/mL increase) | 1.04 | 1.00–1.07 | 0.045 | 1.04 | 1.00–1.07 | 0.044 |
| LV-CMA on PET (continuous value) | 1.00 | 1.00–1.00 | 0.81 | 1.00 | 1.00–1.00 | 0.81 |
| Log LV-CMA on PET | 1.41 | 0.88–2.48 | 0.16 | 1.40 | 0.88–2.46 | 0.17 |
